# Risk Stratification of Cholangiocarcinoma Patients Presenting with Jaundice: A Retrospective Analysis from a Tertiary Referral Center

**DOI:** 10.3390/cancers13092070

**Published:** 2021-04-25

**Authors:** Ana Lleo, Francesca Colapietro, Patrick Maisonneuve, Monia Aloise, Vincenzo Craviotto, Roberto Ceriani, Lorenza Rimassa, Salvatore Badalamenti, Matteo Donadon, Vittorio Pedicini, Alessandro Repici, Luca Di Tommaso, Antonio Voza, Guido Torzilli, Alessio Aghemo

**Affiliations:** 1 Department of Biomedical Sciences, Humanitas University, Pieve Emanuele, 20090 Milan, Italy; francesca.colapietro@humanitas.it (F.C.); lorenza.rimassa@hunimed.eu (L.R.); matteo.donadon@hunimed.eu (M.D.); alessandro.repici@hunimed.eu (A.R.); luca.di_tommaso@hunimed.eu (L.D.T.); antonio.voza@humanitas.it (A.V.); guido.torzilli@hunimed.eu (G.T.); alessio.aghemo@hunimed.eu (A.A.); 2 Internal Medicine Center, IRCCS Humanitas Research Hospital, Rozzano, 20089 Milan, Italy; monia.aloise@humanitas.it (M.A.); vincenzo.craviotto@humanitas.it (V.C.); roberto.ceriani@humanitas.it (R.C.); salvatore.badalamenti@humanitas.it (S.B.); 3 Division of Epidemiology and Biostatistics, IEO European Institute of Oncology IRCCS, 20132 Milan, Italy; patrick.maisonneuve@ieo.it; 4 Medical Oncology and Hematology Unit, Humanitas Cancer Center, IRCCS Humanitas Research Hospital, Rozzano, 20089 Milan, Italy; 5 Divisionof Hepatobiliary and General Surgery, IRCCS Humanitas Research Hospital, Rozzano, 20089 Milan, Italy; 6 Department of Interventional Radiology, IRCCS Humanitas Research Hospital, Rozzano, 20089 Milan, Italy; vittorio.pedicini@humanitas.it; 7 Digestive Endoscopy Unit, IRCCS Humanitas Research Hospital, Rozzano, 20089 Milan, Italy; 8 Pathology Unit, IRCCS Humanitas Research Hospital, Rozzano, 20089 Milan, Italy

**Keywords:** jaundice, cholangiocarcinoma, biliary drainage

## Abstract

**Simple Summary:**

Jaundice is a common clinical presentation of cholangiocarcinoma; however, the prognostic impact of this symptom is poorly understood. We retrospectively analyzed all consecutive cases presenting with jaundice between January 2010 and December 2017. During the study period, 200 patients (0.049% of all admissions) with CCA were identified. Most of them presented with advance disease, and median survival was 4.5 months. Age, stage of disease, presence of jaundice at the moment of diagnosis, and lack of concomitant viral hepatitis were associated with better survival. A nomogram was constructed that significantly predicts short term survival and could be used to tailor management.

**Abstract:**

Cholangiocarcinomas (CCAs) are a heterogeneous group of tumors that arise from the biliary tract. Jaundice is a common clinical presentation; however, the prognostic impact of this symptom is poorly understood, and current management recommendations lack solid evidence. We aim to assess the clinical outcomes and predictive factors of CCA patients presenting with jaundice in the Emergency Room (ER). We retrospectively analyzed all consecutive ER cases presenting with jaundice between January 2010 and December 2017. During the study period, 403,766 patients were admitted to the ER, 1217 (0.3%) presented with jaundice, and in 200 (0.049%), the diagnosis was CCA. CCA cases increased during the study period (*p* for trend 0.026). Most of them presented with advance disease (stage III 46.5%, stage IV 43.5%) and median survival was 4.5 months (95% CI 3.4–6.0). Factors associated with better survival were age, stage of disease, presence of jaundice at the moment of diagnosis, and lack of concomitant viral hepatitis. A nomogram was constructed that significantly predicts 1-month, 6-month, and 1-year survival after patients’ admission. In conclusion, the majority of CCA patients presenting with jaundice to the ER have advanced disease and poor prognosis. Risk stratification of these patients can allow tailored management.

## 1. Introduction

Cholangiocarcinomas (CCAs) are a heterogeneous group of tumors that arise from the biliary tract, and account for nearly 3% of all gastrointestinal tumors [1,2]. CCAs are classified, based on their anatomical origin, as intrahepatic (iCCA), distal (dCCA), perihilar (pCCA), and gallbladder cancer (GBC); pCCA and dCCA are also collectively referred to as extrahepatic (eCCA).

The epidemiologic trend of CCA shows a constant increase in incidence and global mortality worldwide, with the highest incidence reported in Asia where the higher mortality is also observed. CCA incidence currently varies from 85 per 100,000 in northeastern Thailand to 0.4 per 100,000 in Canada [3]. The age-standardized incidence of CCA shows considerable geographical and sex variation, with higher mortality in men than in women [3,4].

Importantly, different subtypes of CCA have diverse risk factors and distinct epidemiological patterns, with global mortality increasing especially in iCCA, while eCCA seems to decrease in most countries [3]. Mortality rates for iCCA have been recently reported to be around 1–2/100,000, whereas for eCCA mortality rates are below 1/100,000 in most countries [3,4,5]. All this evidence might suggest differences in local risk factors and potential genetic predispositions. Importantly, there is still limited international information in large datasets supporting this evidence. Indeed, very limited epidemiological data from single countries with scarce clinical information are available.

Several risk factors have been linked to CCA and, although some of them are shared by all forms of CCA, others seem to be more specific for one subtype [6,7]. However, small case–control studies and a recent meta-analysis are available, but not considering differences in CCA subtypes. A common characteristic among many of these risk factors seems to be that they are associated with chronic damage of the biliary epithelium. Alcohol intake, tobacco smoking, metabolic syndrome, and viral infections—including both hepatitis B virus (HBV) and hepatitis C virus (HCV)—have been reported to increase the risk of CCA [6,8,9]. Further, some biliary diseases characterized by chronic inflammation (i.e., Primary Sclerosing Cholangitis (PSC), and Caroli Disease) are well recognized risk factors for CCA [10,11]. However, the majority of CCA cases often remain sporadic, with no identifiable risk factor present.

Since they are often asymptomatic in early stages, CCAs are usually diagnosed in advanced stages, which highly limits therapeutic options, resulting in a poor prognosis with a 5-year survival of about 5–15% [12], and only 2% in the case of metastasis development. The three CCA subtypes often differ in clinical presentation, natural history, management, and prognosis [1,2,3]. Thus far, there are no well-established non-invasive biomarkers for CCA, although the analysis of the serum levels of the tumor markers CA19-9 and CEA is frequent in the clinical practice in order to help in the diagnosis. In the adjuvant setting, a benefit has been shown with capecitabine [13], with a median overall survival of 53 months in the treatment group and 36 months in the observation group and a significant benefit in progression free survival. Importantly, different CCA molecular subtypes have been again shown to present distinct prognoses and responses to therapy [1]. In recent years, the introduction of next-generation sequencing technologies has allowed a better understanding of the genetic background of CCA; consequently, new treatments tailored to the molecular features or alterations are currently under development.

Jaundice is the most frequent symptom of eCCA due to biliary tract obstruction [14]. In iCCA, jaundice is considered less frequent and mostly associated with advanced disease. Jaundice has been associated to immune dysfunction, increased bacterial translocation, and worsening of nutritional status and liver function. Therefore, in patients with treatable CCA, biliary drainage is recommended to treat obstructive jaundice and optimize the clinical condition before liver surgery or chemotherapy [15,16,17]. Although no solid data are available, biliary drainage is proposed to improve the performance status and survival even of patients that are candidate exclusively to best supportive care. Importantly, the prognostic impact of jaundice in CCA is poorly understood, and current management recommendations lack solid evidence, particularly in patients with advance disease [15].

We therefore aim to assess the clinical outcomes and predictive factors of CCA patients presenting with jaundice in the Emergency Room (ER) of an Academic Italian Hospital.

## 2. Results

### 2.1. Baseline Characteristics

**Figure 1:**
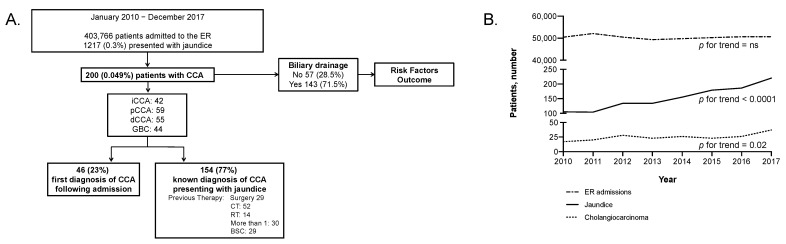
Study flowchart and temporal trends. (**A**). Study flowchart. (**B**). Temporal trends in number of patients with CCA that access the ER.

From January 2010 to December 2017, 403,766 patients were admitted to the ER, 1217 (0.3%) presented with jaundice, and in 200 (0.049%), the diagnosis was CCA (Figure 1A). CCA cases increased from 37 in the 2010–2011 period (37/102,574: 0.036%) to 63 in the 2016–2017 period (63/101,337: 0.062%) (*p* = 0.026) (Figure 1B).

The baseline characteristics of the 200 CCA patients are presented in Table 1.

The median age at the time of ER presentation was 68 years (range 24–88) and 106 (53.0%) patients were male. Extrahepatic CCA was the most common type of cancer (*n* = 114, 57.0%), being pCCA in 59 cases (29.5%) and dCCA in 55 (27.5%); 42 patients (21.0%) presented with iCCA and 44 (22.0%) had GBC.

At the time of presentation in the ER, tumor staging was I-II in 18 (9.0%), III in 93 (46.5%), IV in 87 (43.5%). The majority of patients (161, 80.5%) had a diagnosis of CCA based on histological or cytological evidence; only 34 patients (17.0%) had exclusive radiological diagnosis. The majority of patients (*n* = 154, 77%) had a previous history of CCA, whereas in 46 patients (23.0%) the diagnosis of CCA was triggered by the ER access. Out of the 154 patients that had a known diagnosis of CCA, 29 patients (14.5%) had been resected, whereas 52 had received chemotherapy (26%), 14 (7.0%) had undergone radiotherapy, and 29 (14.5%) had received an indication for best supportive care.

Considering the history of the disease in patients with a previously known diagnosis, jaundice was the most common symptom at diagnosis (*n* = 113, 56.5%), especially in extrahepatic CCA (pCCA *n* = 41, 69.5%; dCCA *n* = 38, 69.1%). Biliary drainage was considered in patients with biliary obstruction and adequate performance status, patients presenting at the ER with jaundice underwent biliary drainage in 71.5% of the cases (*n* = 143). Importantly, none of our patients had PSC, only 10 (5.0%) had cirrhosis, while 35 had diabetes (17.5%).

### 2.2. Outcome and Prognostic Parameters

**Figure 2:**
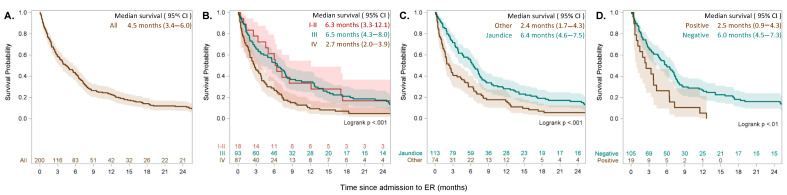
Overall survival of 200 patients with CCA after admission to emergency (**A**) and by selected characteristics (**B**) stage, (**C**) presence of jaundice at disease diagnosis, (**D**) presence or absence of viral hepatitis.

Median survival from the time of enrollment of the 200 patients with CCA included in the study was 4.5 months (95% CI 3.4–6.0). Unexpectedly, the type of CCAs did not impact survival, while age, stage of disease, symptoms at onset, and presence of viral hepatitis were significantly associated with better prognosis (Figure 2, Table 2). Stage IV was associated with a significantly higher rate of mortality (HR 3.87, 95% CI: 1.52–9.84, *p* = 0.005). Similarly, chronic HBV and HCV infection were associated with worse prognosis (HR 2.10, 95% CI: 1.27–3.47, *p* = 0.004). Interestingly, type of jaundice (i.e., obstruction vs. infiltration), liver cirrhosis, diabetes, and obesity were not independent prognostic factors in our study. Clinical symptoms at onset were also associated with better survival in our cohort, with jaundice being associated with a better prognosis (median survival 6.4 months, 95% CI: 4.6–7.5, *p* < 0.001) compared with any other clinical presentation (Figure 2). Of note, the presence of jaundice at diagnosis was associated with better prognosis in all types of CCA independently of the location (Supplementary Figure S1, Table S1). Importantly, 77% of patients in our cohort had a previous CCA diagnosis (Table 1); the presence of symptoms at diagnosis (i.e., jaundice) was an information collected from the medical history. Biliary drainage after ER was not associated with better prognosis at the multivariate analysis (Table 2).

### 2.3. Risk Stratification

**Figure 3:**
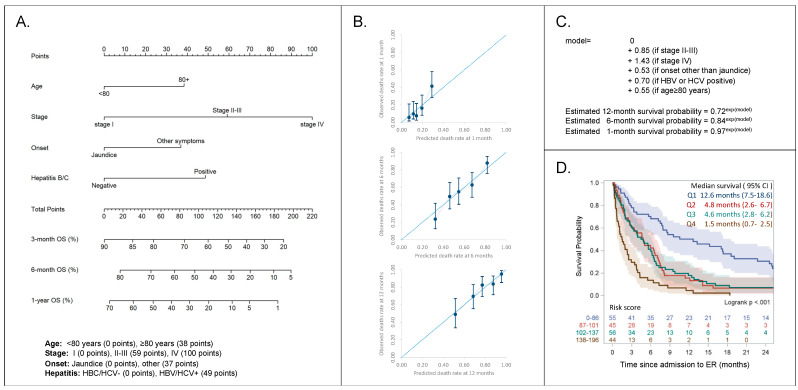
(**A**) Nomogram for the prediction of 3-month, 6-month, and 12-month survival. (**B**) Calibration plots (observed versus expected deaths rate at 1, 6, and 12 months). (**C**) Formula for the calculation of individual survival probability at 1, 6, and 12 months. (**D**) Overall survival of 200 patients with cholangiocarcinoma after admission to emergency by risk score.

A nomogram was constructed based on the results of the final multivariable model aiming to predict 1-month, 6-month, and 1-year survival after patients’ admission to the ER (Figure 3A,B). The formula (Figure 3C) included age (below 80 years old: 0 points, above 80 years: 38 points), stage (I: 0 points, II–III: 59 points, IV: 100 points), symptoms at onset (total bilirubin above 3.5 mg/dl: 0 points, other: 37 points), and viral hepatitis infection (HBC/HCV negative: 0 points, HBV/HCV positive: 49 points). Patients in the lower quartile (total points 0–86) had a significantly better prognosis compared to any other quartile with a median survival of 12.6 months (95% CI 7.5–12.6, *p* < 0.001); on the opposite, patents with a higher score (total points 138–196) had a median survival of 1.5 months (95% CI 0.7–2.5) (Figure 3D). The nomogram was not affected by the type of CCA confirming its reproducibility (Supplementary Figure S2).

## 3. Discussion

CCA is considered a rare tumor and given the lack of large prospective cohorts, very little is known about the natural history. Jaundice is a common symptom in pCCA and dCCA due to biliary obstruction, it is less frequent in iCCA and GBC where is mostly associated with advanced disease. Biliary obstruction is associated to increased risk of cholangitis, biliary pain, and pruritus while also leading to malabsorption and poor nutritional status. Jaundice palliation aims to restore biliary drainage since bilirubin levels are required to be less than twice the upper limit of normal to allow surgery and, in most of the cases, chemotherapeutic agents to be given [18]. Further, biliary drainage has been associated with improved survival and quality of life [15], although no solid data are available, biliary drainage is often proposed to improve the performance status and survival of patients that are candidate exclusively to best supportive care. However, the impact of risks and postprocedural complications (i.e., cholangitis, pancreatitis) have never been assessed in advanced patients. Indeed, current management recommendations lack solid evidence and are often based on the level of local experience.

We herein present a retrospective study aiming to assess the clinical outcomes and predictive factors of CCA patients presenting with jaundice in the ER. Importantly, we included both patients with a previous diagnosis of CCA and patients that were diagnosed after the appearance of jaundice. The majority of patients included in the study (77%) had a previous diagnosis of CCA and had undergone various treatments; however, the aim of the study is not to define natural history, but to identify prognostic factors of CCA patients presenting with jaundice to the ER, besides the previous history of disease. Indeed, the majority (77%; 154/200) of patients included in the study have received different types and scales of treatment. We therefore describe a cohort that is representative of real life and might provide useful information for daily practice. Importantly, the aim of our study is not to provide an insight of the natural history of CCA, but to identify prognostic factors.

We observed that, even if the number of patients presenting to the ER remained relatively constant during the study time, the number of patients with jaundice increased with a slight increase in CCA diagnosis. Patients included in our cohort had poor prognosis with a very short survival (4.5 months); therefore, highlighting the necessity of prognostic tools in order to identify those patients that might benefit from therapy. Surprisingly we did not observe significant differences between eCCA and iCCA, which could be possibly due to the fact that the majority of patients in our cohort presented in a very advanced stage of disease, and almost 30% of them presented with jaundice due to hepatic insufficiency and therefore did not undergo biliary drainage (Table 1). Further, complications after drainage (i.e cholangitis, pancreatitis) might have an impact on survival delaying the onset of specific therapy.

Importantly, age below 80 years, early stage of disease, presence of jaundice at the time of diagnosis, and absence of viral hepatitis were independently and positively associated to better survival at the multivariate analysis. The presence of jaundice at diagnosis could be a surrogate of early diagnosis in extrahepatic CCA, while viral hepatitis might be considered a risk factor for liver disease and impaired liver function. Unfortunately, we could not ascertain the role of antivirals for HBV and HCV infection in our cohort of patients. Still, we think that future research should analyze whether treatment with directly acting antivirals in HCV patients with jaundice and CCA could lead to improvement of liver function and survival. Cirrhosis is known to be a strong risk factor of liver cancer [19], however, in our study it is not associated with survival. However, we must acknowledge that, given the retrospective nature of our study, the information available on cirrhosis was often lacking and the presence of advanced liver disease was inferred through blood parameters and radiological findings while, on the other hand, patients were routinely tested for viral hepatitis. Finally, it is important to highlight that HBsAg positivity, or even more Anti-HCV positivity alone, do not define viral hepatic damage. Surprisingly, our data do not show any significant association between metabolic risk factors and survival, considering that most of the patients presented very late in the natural history of their oncologic disease, data might not be representative of the status of the patients at the time of diagnosis. None of the patients included had a known history of PSC. Importantly, our data confirm for the first time well recognized prognostic factors in a large cohort of CCA patients presenting with jaundice.

Finally, one of the most interesting aspects of our study was the development of a nomogram that allows us to stratify patients based on rapidly available prognostic factors. Importantly, patients on the first quartile with a score 0–86 present a significantly better prognosis (survival 12.6 months) compared to the others (Figure 3), while biliary drainage after ER was not associated with better prognosis at the multivariate analysis (Table 2). This tool allows, for the first time, to identify CCA jaundiced patients that could benefit from treatment and in the future could also be used to stratify and homogenize patients at baseline in clinical trials or interventional studies.

Our study presents a number of strengths and weaknesses that are worth mentioning. Among the former, this is the first study that aims to study the prognosis and risk factors of CCA patients presenting with jaundice to the ER; given the fact that incidence and mortality of this disease is increasing [3,20], this information is relevant for the clinical management of CCA patients. Second, the sample size of this study is significant; we indeed include 200 patients with CCA presenting with jaundice. Third, the study was performed in a single center, which guarantees a uniform clinical management of patients in terms of indication for biliary drainage or other medical treatment. Fourth, this is the first study providing a tool for risk stratification that allows to identify patients with a significantly better survival.

Among weaknesses, the retrospective design of the study is predominant, but we should also be aware that the numerosity of the study population and long study period support our conclusions. Second, this was a single center study of a tertiary referral center, which might be not representative of most of the centers. Third, our data might be biased due to the fact that only patients with a very long history of disease presented to the ER, and the majority of patients had advance disease. Fourth, the presented nomogram lacks external validation.

In conclusion, the present study—although limited by its retrospective nature and the lack of validation—gives some interesting insights that might help managing CCA patients presenting with jaundice. Our data show indeed that the majority of CCA patients presenting with jaundice to the ER have advanced disease and poor prognosis, and that risk stratification of these patients can allow tailored management.

## 4. Materials and Methods

### 4.1. Study Design and Participants

We retrospectively analyzed all adult patients consecutively admitted with jaundice to the ER of Humanitas Research Hospital between January 2010 and December 2017. Jaundice was defined as total bilirubin equal or higher than 3.5 mg/dL. All jaundiced patients, with both new and previously diagnosed CCA, and with an overall follow-up of at least 24 months, were included in the analysis.

Diagnosis of CCA was defined according to both histological/cytological or radiological evaluation. Stage was established according to the AJCC 8th edition staging system, considering site of disease [21].

The primary endpoint of the study was overall survival (OS), calculated from the date of admission to the ER to the date of death or the date of last assessment of vital status. Demographic, clinical, laboratory, and outcome data were obtained from electronic medical records and were checked by three expert physicians. Patient demographic and clinicopathologic data at the time of ER presentation included age, sex, date of CCA diagnosis, symptoms of CCA onset, site of disease (iCCA, dCCA, pCCA, and GBC), stage, previous treatment, cause of jaundice (obstructive versus infiltration), and biliary drainage for jaundice palliation (including both endoscopic and percutaneous procedures). Risk factors as lithiasis, hepatitis (i.e., HCV or HBV), alcohol intake, cirrhosis, and metabolic comorbidities (i.e., hypertension, diabetes, obesity) we also included. The diagnosis of cirrhosis was made using a combination of both imaging (CT scan) and biochemical tests. In particular, we used extensively validated scores as Fibrosis-4 (FIB-4), that includes age, transaminases, and platelets count; FIB-4 above 3.25 has a 97% specificity and a positive predictive value of 65% for cirrhosis [22]). Further, all patients’ radiological documentation was revised by two independent radiologists. The data cut-off was 31 December 2019.

The study was approved by the local Ethical Committee. Specific informed consent was waived because this study used deidentified retrospective data; however, all patients signed a consent form allowing the use of retrospective data at the time of admission. This report follows the Strengthening the Reporting of Observational Studies in Epidemiology (STROBE) reporting guideline [23].

### 4.2. Statistical Methods

OS curves were plotted using the Kaplan–Meier methods and survival between groups was compared with the Log-rank test. Univariable Cox proportional Hazard’s regression was used to identify factors associated with OS. Variables significantly associated with OS at univariate analysis were entered in a multivariable model and backward stepwise selection was used to retain independent predictors of OS. A nomogram was constructed based on the results of the final multivariable model to predict 1-month, 6-month, and 1-year OS after patients’ admission to emergency. The accuracy of the multivariable models was assessed visually with calibration curves plotting the predicted against the observed survival in groups of patients defined by quantiles of predicted probabilities. Statistical analysis was performed using SAS software version 9.4 (SAS Institute, Cary, NC, USA) and the R 3.4.4 packages rms and Hmisc. Statistical significance was defined by a two-tailed *p* value < 0.05.

## 5. Conclusions

Our data from a large retrospective study support that CCA patients presenting to the ER with jaundice have advance disease and poor prognosis. Risk stratification in these patients is mandatory and can be established based on age, stage of disease, symptoms at onset, and presence of viral hepatitis, allowing a better and tailored management of these patients.

## Figures and Tables

**Table 1:** Patients’ characteristics at the time of ER presentation.

Baseline Characteristics	All *n* (%)	iCCA *n* (%)	pCCA *n* (%)	dCCA *n* (%)	GBC *n* (%)
All	200 (100)	42 (100)	59 (100)	55 (100)	44 (100)
Gender					
Male	106 (53.0)	19 (45.2)	35 (59.3)	30 (54.5)	22 (50.0)
Female	94 (47.0)	23 (54.8)	24 (40.7)	25 (45.5)	22 (50.0)
Age					
<50	12 (6.0)	5 (11.9)	4 (6.8)	3 (5.5)	-
50–59	25 (12.5)	5 (11.9)	7 (11.9)	6 (10.9)	7 (15.9)
60–69	63 (31.5)	16 (38.1)	18 (30.5)	13 (23.6)	16 (36.4)
70–79	70 (35.0)	11 (26.2)	24 (40.7)	20 (36.4)	15 (34.1)
80+	30 (15.0)	5 (11.9)	6 (10.2)	13 (23.6)	6 (13.6)
Bilirubin, mean ± SD (mg/dL)	12.4 ± 3.0	10.2 ± 5.5	12.7 ± 2.5	17.3 ± 2.5	9.7 ± 1.7
Ca19.9, mean ± SD (IU/mL)	995 ± 219	892 ± 102	1023 ± 285	945 ± 382	1123 ± 107
CEA, mean ± SD (ng/mL)	25 ± 19	24 ± 12	32 ± 27	29 ± 18	18 ± 11
Stage					
I	6 (3.0)	2 (4.8)	2 (3.4)	-	2 (4.5)
II	12 (6.0)	1 (2.4)	7 (11.9)	3 (5.5)	1 (2.3)
III	93 (46.5)	16 (38.1)	33 (55.9)	31 (56.4)	13 (29.5)
IV	87 (43.5)	23 (54.8)	16 (27.1)	21 (38.2)	27 (61.4)
N/a	2 (1.0)	-	1 (1.7)	-	1 (2.3)
Diagnosis of CCA					
First diagnosis	46 (23.0)	4 (9.5)	19 (32.2)	13 (23.6)	10 (22.7)
Previously known CCA	154 (77.0)	38 (90.5)	40 (67.8)	42 (76.4)	34 (77.3)
If previous, treatment *					
Surgery	29 (14.5)	8 (19.0)	3 (5.1)	7 (12.7)	11 (25.0)
CT	52 (26.0)	17 (40.5)	8 (13.6)	13 (23.6)	14 (31.8)
RT	14 (7.0)	3 (7.1)	3 (5.1)	5 (9.1)	3 (6.8)
Best Supportive Care	29 (14.5)	6 (14.3)	8 (13.6)	4 (7.3)	11 (25.0)
Onset					
Jaundice (bilirubin > 3.5 mg/dL)	113 (56.5)	11 (26.2)	41 (69.5)	38 (69.1)	23 (52.3)
Laboratory	27 (13.5)	14 (33.3)	5 (8.5)	1 (1.8)	7 (15.9)
Weight loss	11 (5.5)	2 (4.8)	5 (8.5)	1 (1.8)	3 (6.8)
Cholangitis	12 (6.0)	1 (2.4)	3 (5.1)	4 (7.3)	4 (9.1)
Other	37 (18.5)	14 (33.3)	5 (8.5)	11 (20)	7 (15.9)
Lithiasis					
No	132 (66.0)	34 (81)	47 (79.7)	33(60.0)	18 (40.9)
Yes	68 (34.0)	8 (19.0)	12 (20.3)	22 (40.0)	26 (59.1)
HCV-Ab or HBsAg					
Negative	105 (52.5)	21 (50.0)	31 (52.5)	32 (58.2)	21 (47.7)
Positive	19 (9.5)	11 (26.2)	4 (6.8)	3 (5.5)	1 (2.3)
N/a	76 (38.0)	10 (23.8)	24 (40.7)	20 (36.4)	22 (50.0)
Alcohol					
No	187 (93.5)	39 (92.9)	57 (96.6)	51 (92.7)	40 (90.9)
Yes	13 (6.5)	3 (7.1)	2 (3.4)	4 (7.3)	4 (9.1)
Comorbidities					
Hypertension	92 (46.0)	17 (40.5)	33 (55.9)	20 (36.4)	22 (50.0)
Cirrhosis	10 (5.0)	6 (14.3)	1 (1.7)	1 (1.8)	2 (4.5)
Diabetes	35 (17.5)	8 (19.0)	10 (16.9)	9 (16.4)	8 (18.2)
Obesity	20 (10)	4 (9.5)	3 (5.1)	5 (9.1)	8 (18.2)
Histology					
No	39 (19.5)	6 (14.3)	18 (30.5)	7 (12.8)	8 (18.2)
Yes	161 (80.5)	36 (85.7)	41 (69.5)	48 (87.3)	36 (81.8)
Biliary drainage after ER					
No	57 (28.5)	23 (54.8)	11 (18.6)	14 (25.5)	9 (20.5)
Yes	143 (71.5)	19 (45.2)	48 (81.4)	41 (74.5)	35 (79.5)
If yes, type of drainage					
PTBD	60 (41.9)	16 (84.2)	9 (18.7)	3 (7.3)	32 (91.4)
Endoscopy	83 (58.1)	3 (15.8)	39 (81.3)	38 (92.7)	3 (8.6)

* A patient may have received more than one type of treatment.

**Table 2:** Factors associated with mortality.

Variable	Comparison Groups	Univariable Analysis		Multivariable Analysis	
		HR (95% CI)	*p*-Value	HR (95% CI)	*p*-Value
Site	Extra-hepatic (vs. iCCA)	0.77 (0.53–1.11)	0.16		
	Gallbladder (vs. iCCA)	1.13 (0.73–1.74)	0.57		
Gender	Female (vs. male)	1.21 (0.91–1.61)	0.19		
Age	≥80 years (vs. <80 years)	1.32 (0.89–1.96)	0.17	1.73 (1.15–2.62)	0.009
Stage	II (vs. I)	2.28 (0.78–6.72)	0.13	2.67 (0.88–8.05)	0.08
	III (vs. I)	2.06 (0.81–5.20)	0.13	2.31 (0.89–5.95)	0.08
	IV (vs. I)	3.87 (1.52–9.84)	0.005	4.20 (1.61–11.0)	0.003
First presentation	Yes (vs. no)	0.75 (0.53–1.06)	0.10		
Previous Surgery	Yes (vs. no)	1.13 (0.75–1.69)	0.57		
Previous CT	Yes (vs. no)	1.27 (0.91–1.76)	0.16		
Previous RT	Yes (vs. no)	1.91 (1.10–3.30)	0.02		
Previous palliative	Yes (vs. no)	1.46 (0.96–2.20)	0.07		
Disease onset	Other (vs. jaundice)	1.72 (1.27–2.33)	0.0004	1.71 (1.25–2.33)	0.0008
Lithiasis	Yes (vs. no)	0.99 (0.73–1.35)	0.96		
Hepatitis	Positive (vs. negative)	2.10 (1.27–3.47)	0.004	2.00 (1.20–3.33)	0.008
Alcohol	Yes (vs. no)	0.65 (0.36–1.18)	0.15		
Body mass index	Overweight (vs. normal)	0.85 (0.41–1.79)	0.68		
	Obese (vs. normal)	0.50 (0.20–1.30)	0.16		
Hypertension	Yes (vs. no)	0.89 (0.66–1.19)	0.42		
Cirrhosis	Yes (vs. no)	1.08 (0.57–2.05)	0.82		
Diabetes	Yes (vs. no)	0.98 (0.68–1.43)	0.93		
Histology	CTM (vs. histology)	1.21 (0.70–2.10)	0.50		
	No (vs. histology)	1.40 (0.96–2.06)	0.08		
Biliary drainage after ER	Yes (vs. no)	0.73 (0.53–1.01)	0.05		

CT: chemotherapy, RT: radiotherapy, BSC: best supportive care, HR: hazard ratio.

## Data Availability

The data presented in this study are available on request from the corresponding author. The data are not publicly available due to privacy.

## Supplementary Materials

The following are available online at https://www.mdpi.com/article/10.3390/cancers13092070/s1, Figure S1: Overall survival after admission by CCA type, Figure S2: Overall survival by risk score and CCA type, Table S1: Multivariate analysis separated by the type of CCA.
