# Supplementary material for: Risk Stratification of Cholangiocarcinoma Patients Presenting with Jaundice: A Retrospective Analysis from a Tertiary Referral Center
Source: Cancers (Basel). 2021 Apr 25;13(9):2070. doi: 10.3390/cancers13092070 (PMC8123266; doi:10.3390/cancers13092070)
Supplement: Supplementary file 1 [file cancers-13-02070-s001.zip › cancers-1179551-supplementary.pdf]

Risk Stratification of Cholangiocarcinoma Patients Presenting with Jaundice: A Retrospective Analysis from a Tertiary Referral center

Ana Lleo, Francesca Colapietro, Patrick Maisonneuve, Monia Aloise, Vincenzo Craviotto, Roberto Ceriani, Lorenza Rimassa, Salvatore Badalamenti, Matteo Donadon, Vittorio Pedicini, Alessandro Repici, Luca Di Tommaso, Antonio Voza, Guido Torzilli and Alessio Aghemo

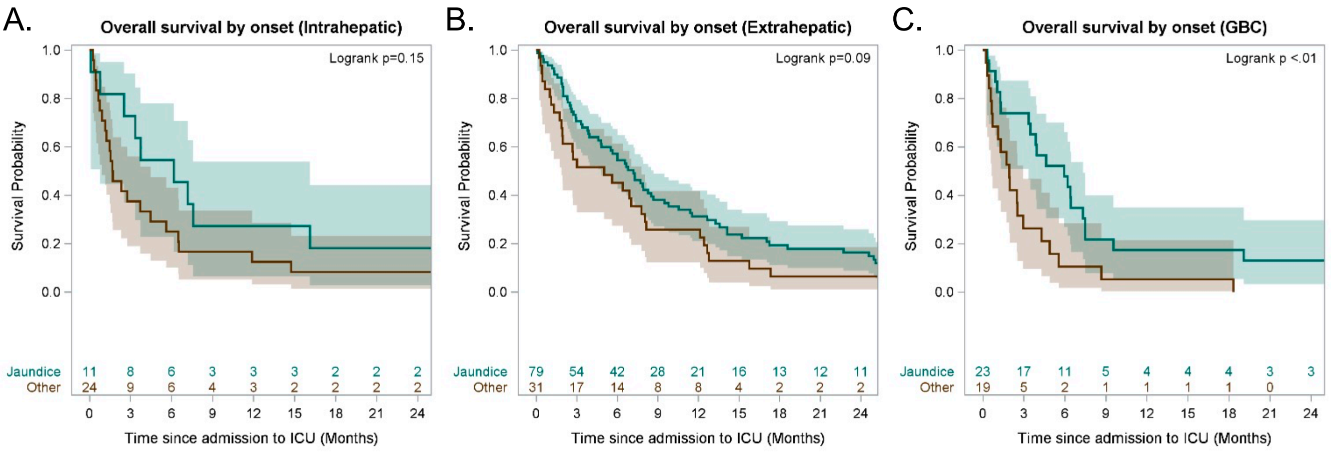

Figure S1. Overall survival of 200 patients with iCCA (A), eCCA (B), and GBC (C) after admission to emergency by presence of jaundice at disease diagnosis.

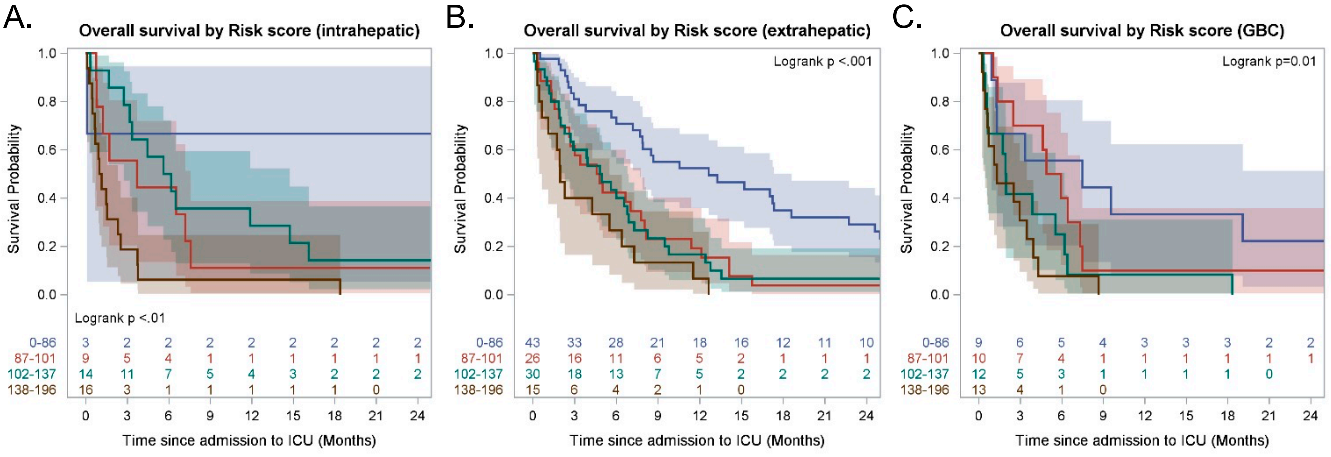

Figure S2. Overall survival of 200 patients with iCCA (A), eCCA (B), and GBC (C) risk score.

Table S1. Factors associated with mortality. Multivariate analysis separated by the type of CCA regarding the location of the tumor (i.e., iCCA, eCCA, and GBC).

		**iCCA (*n* = 42)**		**eCCA (*n* = 114)**		**GBC (*n* = 44)**	
**Variable**	**Comparison Groups**	**HR (95% CI)**	***p*-value**	**HR (95% CI)**	***p* -value**	**HR (95% CI)**	***p* -value**
Age	≥80 years (vs <80 years)	2.05 (0.70–6.03)	0.19	1.57 (0.89–2.75)	0.12	2.49 (0.81–7.62)	0.11
Stage	II (vs I)	-		2.04 (0.41–10.2)	0.39	-	
	III (vs I)	6.75 (0.70–65.0)	0.10	1.64 (0.38–7.13)	0.51	0.25 (0.02–3.03)	0.28
	IV (vs I)	6.73 (0.81–55.9)	0.08	3.73 (0.83–16.8)	0.09	0.48 (0.04–5.35)	0.55

Disease onset	Other (vs jaundice)	1.46 (0.58–3.67)	0.42	1.45 (0.92–2.30)	0.11	2.23 (1.00–5.01)	0.05
Hepatitis	Positive (vs negative)	3.54 (1.38–9.10)	0.009	1.14 (0.49–2.62)	0.76	0.19 (0.07–5.00)	0.32
